# Metabolomics-Based Identification of α-Glucosidase Inhibitors from *Pometia pinnata* Stem Bark Using LC-HRMS and Molecular Docking

**DOI:** 10.3390/molecules31183233

**Published:** 2026-09-13

**Authors:** Husniati Husniati, Berna Elya, Muhammad Hanafi, Puspa Dewi Narrij Lotulung, Faris Hermawan, Rifaldi Rifaldi, Dela Rosa, Alfi Khatib

**Affiliations:** 1 Department of Pharmacognosy and Phytochemistry, Faculty of Pharmacy, Universitas Indonesia, Depok 16424, Indonesia; husn009@brin.go.id; 2 Research Center for Pharmaceutical Ingredients and Traditional Medicine, National Research and Innovation Agency, KST BJ Habibie, South Tangerang 15314, Indonesia; muha002@brin.go.id (M.H.); pusp001@brin.go.id (P.D.N.L.); fari025@brin.go.id (F.H.); rifa006@brin.go.id (R.R.); 3 Faculty of Pharmacy, Universitas Pancasila, Jakarta 12640, Indonesia; 4 Department of Pharmacy, Faculty of Health Sciences, Universitas Pelita Harapan, Tangerang 15811, Indonesia; dela.rosa@uph.edu; 5 Department of Pharmaceutical Chemistry, Faculty of Pharmacy, International Islamic University Malaysia, Kuantan 25200, Malaysia; alfikhatib@iium.edu.my; 6 Faculty of Pharmacy, Universitas Airlangga, Surabaya 60115, Indonesia

**Keywords:** *Pometia pinnata*, α-glucosidase inhibitor, untargeted metabolomics, LC-HRMS, molecular docking

## Abstract

*Pometia pinnata* J.R. Forst. & G. Forst. is traditionally used throughout tropical Asia and the Pacific to manage diabetes-associated hyperglycemia. However, the metabolites responsible for its α-glucosidase inhibitory activity (AGI) remain poorly characterized. This study aimed to identify putative AGI-associated metabolites from *P. pinnata* stem bark through metabolomics-based prioritization and tentative annotation using untargeted LC–HRMS, followed by molecular docking to assess their interactions with α-glucosidase. Thirty ethyl acetate–methanol gradient fractions were analyzed by orthogonal partial least squares (OPLS) to prioritize LC–HRMS features associated with AGI activity, followed by molecular docking of the tentatively annotated metabolites against *Saccharomyces cerevisiae* α-glucosidase (3A4A) and human maltase-glucoamylase (3TOP). The 75% ethyl acetate in methanol fraction showed the strongest AGI, with an IC_50_ of 5.53 μg/mL. Five AGI-associated metabolites, namely scopoletin, 3,4-dihydroxybenzaldehyde, fisetin, 4-methoxycinnamic acid, and lindetannin, were tentatively annotated in *P. pinnata* stem bark based on LC-HRMS/MS data. To the best of our knowledge, these annotations have not previously been reported in *P. pinnata* stem bark. Among these candidates, fisetin showed the most favorable binding interactions with both target enzymes. Metabolomics-guided isolation, followed by NMR analysis, confirmed the structure of scopoletin, although the isolated compound showed weak AGI activity (IC_50_ > 200 μg/mL). The marked difference between the parent fraction and isolated scopoletin indicates that scopoletin alone is unlikely to account for the observed activity and that other constituents may contribute. Nevertheless, this study provides a promising metabolomics-guided framework for prioritizing and tentatively annotating candidate AGI-associated metabolites in the stem bark of *P. pinnata*.

## 1. Introduction

Hyperglycemia is a hallmark of type 2 diabetes mellitus (T2DM), which accounts for about 90% of all diabetes cases and imposes a significant burden on healthcare systems [1]. The number of people with diabetes has increased quickly worldwide and may reach about 900 million by 2050 [2]. One way to control high blood sugar after meals is by blocking α-glucosidase, an enzyme in the intestine that breaks down carbohydrates [3]. Drugs such as acarbose, miglitol, and voglibose can cause gastrointestinal side effects [4,5], prompting ongoing efforts to identify safer, more effective natural alternatives [6].

*Pometia pinnata* J.R. Forst. & G. Forst. (Sapindaceae) is a medicinal plant widely distributed throughout tropical Asia and the Pacific region, and has attracted considerable attention for its antihyperglycemic potential [7]. Previous studies have demonstrated the AGI activity of this species. Phytochemical investigations of the leaves have led to the isolation of several bioactive constituents. Utari et al. [8] isolated and identified two major flavonol rhamnosides, kaempferol-3-O-rhamnoside (afzelin) and quercetin-3-O-rhamnoside (quercitrin), from the leaf ethyl acetate fraction, both of which exhibited AGI activity. More recently, Istiqamah et al. [9] isolated proanthocyanidin A2 from the leaves and demonstrated that it was the most potent AGI among the isolated compounds. Recently, LC–HRMS profiling of the ethyl acetate extract of the stem bark identified 26 metabolites, with cianidanol emerging as the most promising candidate for AGI activity, based on metabolite profiling and molecular docking analysis [10]. Despite these advances, only a limited number of bioactive constituents have been characterized from the stem bark. Therefore, a comprehensive metabolomics-guided strategy is needed to facilitate the discovery of additional metabolites responsible for its α-glucosidase inhibitory activity.

Metabolomics has emerged as a powerful approach for comprehensively characterizing the chemical composition of natural products and correlating metabolite profiles with biological activities. Integrating high-resolution analytical platforms with bioactivity data enables rapid prioritization of candidate bioactive metabolites and provides insights into the potential contributions of multiple metabolites to the observed biological effects [11]. However, the complexity of untargeted metabolomics datasets necessitates the use of robust multivariate statistical tools to identify metabolites associated with specific biological responses. Orthogonal partial least squares (OPLS) analysis has been widely adopted for this purpose and has proven effective in metabolomics-guided natural product discovery [12]. This strategy has successfully facilitated the identification of bioactive metabolites with antidiabetic, anti-HIV, and anticholinesterase activities from diverse medicinal plants [13,14,15].

Despite these promising findings, the metabolites responsible for AGI of *P. pinnata* stem bark have not been comprehensively characterized. Untargeted metabolomics remains necessary to accelerate the identification of bioactive metabolites through LC–HRMS-based profiling. Therefore, this study aimed to identify putative AGI-associated metabolites from *P. pinnata* stem bark through metabolomics-based prioritization and tentative annotation using untargeted LC–HRMS, followed by molecular docking and metabolomics-guided isolation. We hypothesized that the AGI activity of *P. pinnata* stem bark is associated with specific metabolites that can be prioritized based on their statistical association with bioactivity, tentatively annotated through LC–HRMS/MS analysis, and further evaluated for their potential interactions with α-glucosidase and, where possible, structurally validated through metabolomics-guided isolation. This integrated strategy is expected to provide a systematic framework for the metabolomics-based prioritization and tentative annotation of candidate AGI-associated metabolites, while guiding their subsequent structural characterization and evaluation as potential antidiabetic agents.

## 2. Results

### 2.1. AGI Activity

Table 1 shows the AGI activities of the fractions collected using the ethyl acetate and methanol solvent gradient. All fractions inhibited α-glucosidase, with IC_50_ values ranging from 5.53 ± 0.70 to 10.77 ± 1.24 μg/mL. The 75% ethyl acetate in methanol fraction had the lowest IC_50_ value at 5.53 ± 0.70 μg/mL, followed by the 50% ethyl acetate in methanol fraction at 6.95 ± 0.50 μg/mL. The pure methanol fraction had an IC_50_ of 8.34 ± 0.65 μg/mL. The 100% ethyl acetate and 25% ethyl acetate in methanol fractions had IC_50_ values of 10.68 ± 1.18 and 10.77 ± 1.24 μg/mL, respectively. Most fractions showed significant differences (*p* < 0.05), but the activities of the 75% and 50% ethyl acetate in methanol fractions were not significantly different.

### 2.2. Metabolomics Analysis

LC–HRMS analysis of the 30 fractions generated a dataset comprising 1947 metabolite features, each defined by its retention time and *m*/*z* value. OPLS was then used to link these metabolite profiles with α-glucosidase inhibitory activity (1/IC_50_ AGI). The resulting model contained two predictive components and yielded cumulative R^2^*_X_*, R^2^*_Y_*, and Q^2^ values of 0.56, 0.601, and 0.435, as shown in Table 2.

**Figure 1:**
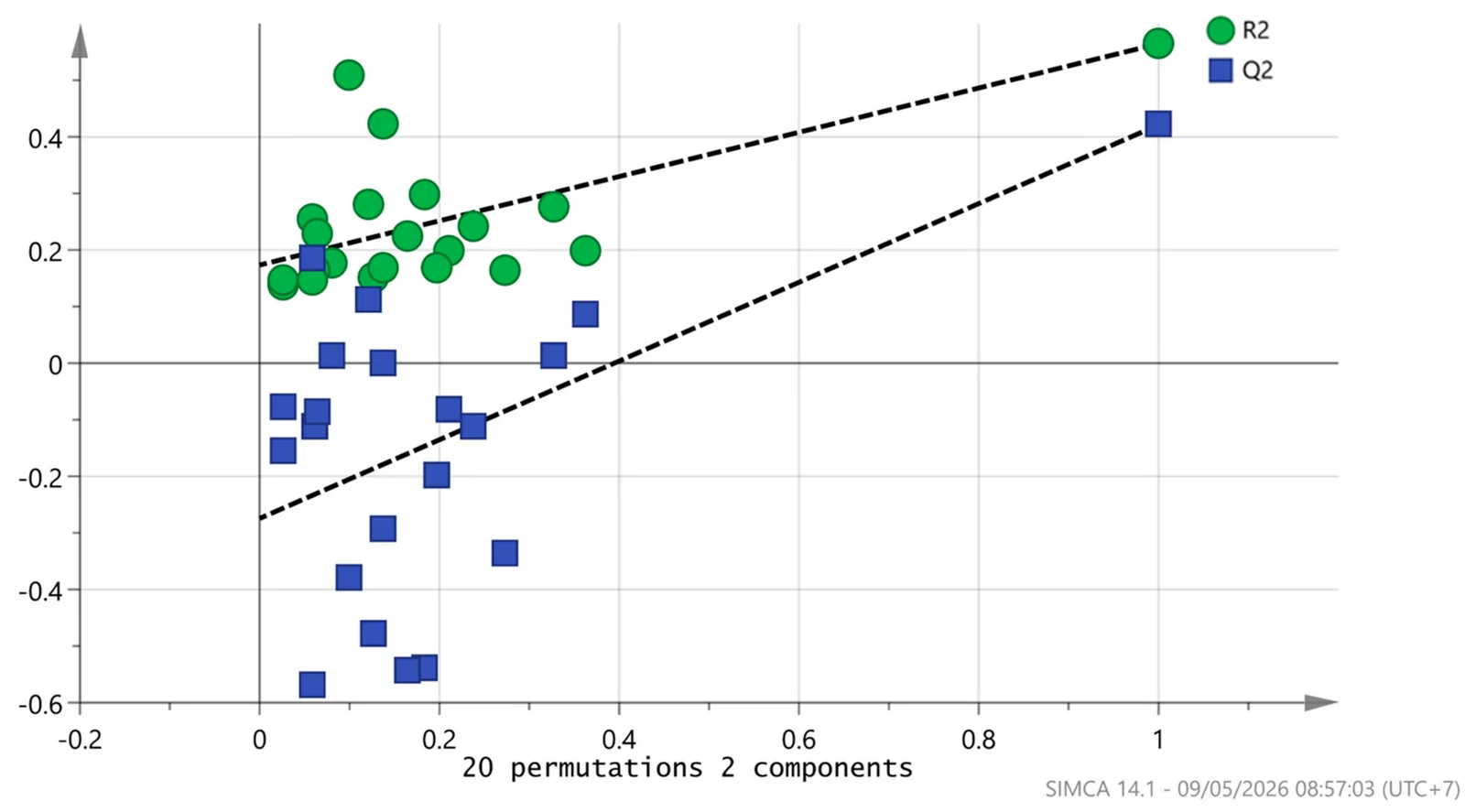
OPLS permutation test (20 permutations) for the model linking LC–HRMS metabolite profiles of *Pometia pinnata* stem bark fractions with α-glucosidase inhibitory activity (1/IC_50_ AGI). The x-axis shows the correlation coefficient between the original and permuted response variables, and the y-axis shows the corresponding R^2^ and Q^2^ values. Dashed lines represent the regression lines for the R^2^ and Q^2^ values obtained from the permutation tests.

The validity of the OPLS model was evaluated using a 20-permutation test (Figure 1). The original model yielded R^2^ = 0.65 and Q^2^ = 0.45, whereas the permuted models consistently yielded lower values, supporting the observed relationship. The regression intercepts for the permuted models were R^2^ = 0.273 and Q^2^ = −0.336.

CV-ANOVA was used to assess the statistical significance of the OPLS model based on cross-validated predictive residuals. The model yielded a *p*-value of 0.0084, below the significance threshold of 0.05, indicating statistical significance.

**Figure 2:**
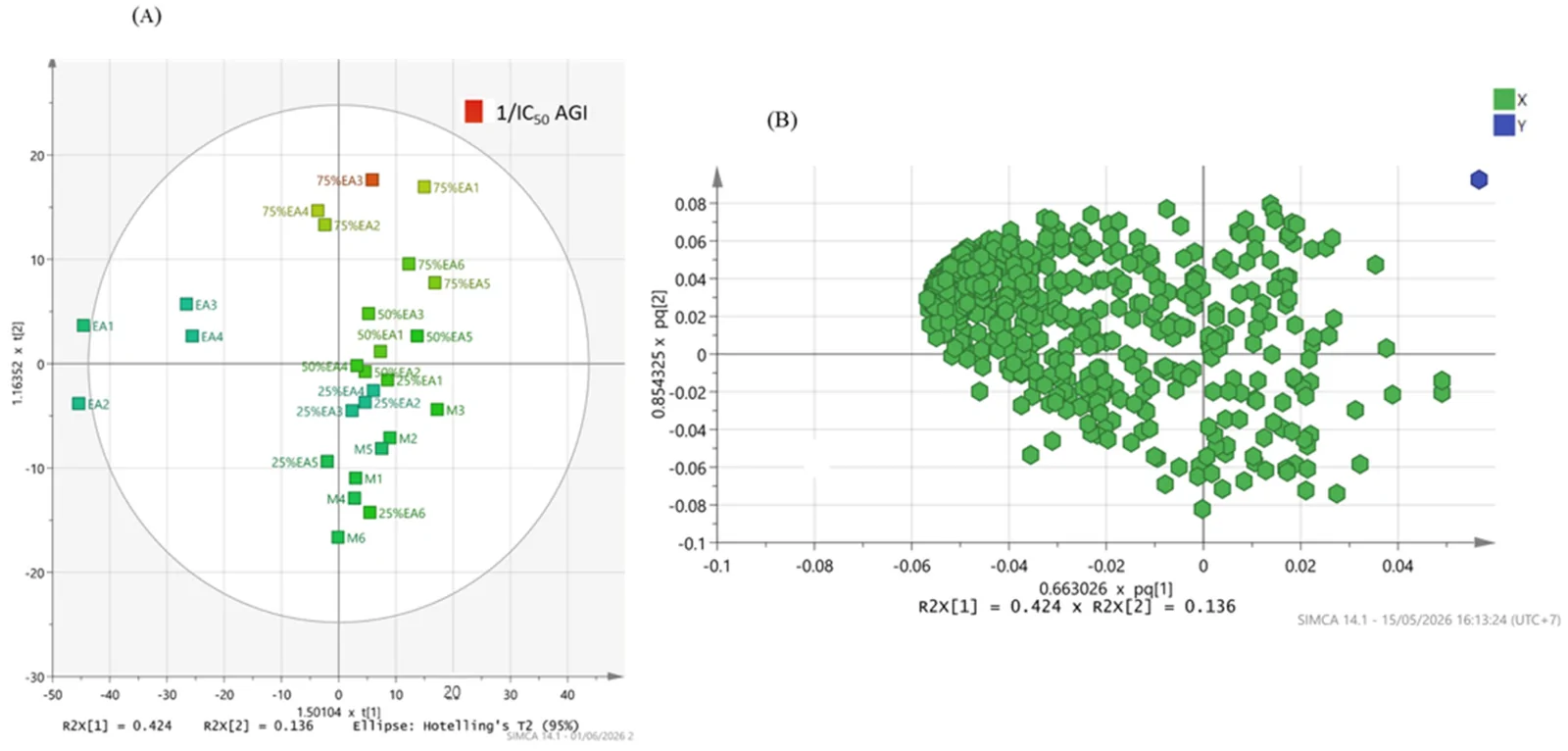
OPLS score and loading scatter plots showing the relationship between LC–HRMS metabolite profiles of *Pometia pinnata* stem bark fractions and α-glucosidase inhibitory activity (1/IC_50_ AGI). In panel (**A**), each point on the OPLS score plot represents a single fraction. M1–M6 are 100% methanol fractions, 25%EA1–25%EA6 are 25% ethyl acetate in methanol fractions, 50%EA1–50%EA6 are 50% ethyl acetate in methanol fractions, 75%EA1–75%EA6 are 75% ethyl acetate in methanol fractions, and EA1–EA6 are 100% ethyl acetate fractions. The numbers 1–6 show six separate replicate fractions made from each solvent mixture. The colors and symbols distinguish the corresponding fraction groups, while the red square represents the 1/IC_50_ AGI response. Panel (**B**) shows the OPLS loading scatter plot, which displays the LC–HRMS features that contribute to the predictive component. Green symbols represent X variables, whereas blue symbols represent Y variables.

The OPLS score plot (Figure 2A) shows the distribution of the 30 fraction samples based on their LC–HRMS metabolite profiles and (1/IC_50_) AGI activity. Replicate fractions prepared with the same solvent composition generally clustered closely, indicating similar metabolite profiles among replicates. Fractions obtained with 75% and 50% ethyl acetate in methanol were predominantly distributed on the positive side of the predictive component, whereas the 100% ethyl acetate fractions were mainly located on the negative side. Fractions obtained with the other solvent compositions occupied intermediate positions within the score plot.

The loading scatter plot in Figure 2B shows the distribution of LC–HRMS features in the predictive part of the OPLS model. Variable-importance analysis identified 14 LC–HRMS features with VIP > 1, which were therefore selected for further study (see Table 3). These features appeared at retention times between 4.53 and 18.30 min and had *m*/*z* values from 139.04 to 865.20. Their VIP values ranged from 1.04 to 1.38. The *pq*[1] values ranged from 0.0382 to 0.0569, and the *pq*[2] values ranged from 0.0234 to 0.0559. The *pq*[1] and *pq*[2] values indicate the direction and magnitude of each variable’s contribution to the predictive component of the OPLS model.

### 2.3. Metabolite Annotation

After selecting features, the prioritized features were evaluated for metabolite annotation using accurate-mass measurements and MS/MS fragmentation spectra acquired in positive ion mode. Molecular formulas were assigned from the accurate-mass measurements, and candidate structures were assessed by comparing the observed MS/MS fragmentation patterns with reference spectra available in mzCloud™ and ChemSpider™. Based on the available LC–HRMS/MS evidence, five of the 14 prioritized features were tentatively annotated as scopoletin, 3,4-dihydroxybenzaldehyde, fisetin, 4-methoxycinnamic acid, and lindetannin. These annotations were classified as MSI Level 2 because they were supported by accurate-mass measurements and MS/MS spectral-library evidence but were not confirmed against authentic reference standards (Supplementary Materials Table S1). The remaining nine features could not be reliably annotated because the available MS/MS evidence and database matches were insufficient to support specific metabolite assignments. Table 4 summarizes the five tentative annotations. All five showed low mass errors and MS/MS fragmentation patterns consistent with the proposed metabolite assignments.

Scopoletin, a coumarin-derived phenolic compound, was tentatively identified by its protonated molecular ion at *m*/*z* 193.0498 ([M+H]^+^), corresponding to the molecular formula C_10_H_8_O_4_. Under positive electrospray ionization, the precursor ion underwent characteristic high-energy collision dissociation (HCD), initially generating a fragment ion at *m*/*z* 178.0263 through the loss of a methyl radical (•CH_3_) from the methoxy substituent. The resulting ion remained stabilized by resonance across the conjugated coumarin framework. An alternative fragmentation pathway involved the neutral loss of C_2_H_4_O_2_, yielding a diagnostic product ion at *m*/*z* 133.0283 corresponding to a resonance-stabilized 6-hydroxybenzofuran-5-yl cation. These observed product ions were consistent with previously reported MS/MS characteristics of scopoletin, providing additional support for its tentative annotation [16].

The MS/MS spectrum of 3,4-dihydroxybenzaldehyde showed a protonated molecular ion at *m*/*z* 139.0387 ([M+H]^+^). Upon HCD activation, the precursor ion first underwent decarbonylation through the neutral loss of CO from the aldehyde moiety, yielding a prominent fragment ion at *m*/*z* 111.0441 ([M+H−CO]^+^). Subsequent dehydration generated a product ion at *m*/*z* 93.0337 ([M+H−CO−H_2_O]^+^), reflecting further rearrangement of the aromatic structure. A product ion at *m*/*z* 93 has also been reported for 3,4-dihydroxybenzaldehyde in previous LC–MS/MS analysis, providing supporting spectral evidence for the proposed annotation [17].

Fisetin (C_15_H_10_O_6_) produced a protonated molecular ion at *m*/*z* 287.0547. Collision-induced fragmentation predominantly involved cleavage of the heterocyclic C ring, accompanied by the neutral loss of C_8_H_6_O_3_, yielding a characteristic product ion at *m*/*z* 137.0233. This fragment corresponds to a resonance-stabilized benzopyrylium ion derived from the oxygen-containing A ring and is a typical fragmentation feature of flavonols under positive-ion MS/MS conditions. A related product ion at *m*/*z* 135 has been reported for fisetin under negative-electrospray conditions [18]. Because the reported spectrum was acquired under a different ionization mode and the study was primarily focused on metabolite identification, the literature result is considered supporting evidence for the metabolite annotation rather than direct evidence for the fragmentation mechanism observed here.

4-Methoxycinnamic acid generated a protonated molecular ion at *m*/*z* 179.0700 ([M+H]^+^), consistent with the molecular formula C_10_H_10_O_3_. In the present HCD-MS/MS spectrum, product ions at *m*/*z* 123.0440 and 105.0335 were observed, consistent with sequential neutral losses of C_3_H_4_O and H_2_O, respectively. These fragmentation features were therefore used as supporting evidence for the tentative annotation of 4-methoxycinnamic acid.

Lindetannin was tentatively assigned based on its protonated molecular ion at *m*/*z* 865.1954 ([M+H]^+^), corresponding to the molecular formula C_45_H_36_O_18_. Its MS/MS spectrum showed several product ions, including *m*/*z* 533.1073, 123.0440, 287.0546, and 257.0441, indicating extensive fragmentation of the condensed tannin structure. The observed fragmentation pattern was considered consistent with proanthocyanidin-type tannins. Supporting evidence was provided by Wang et al. [19], who reported lindetannin with a characteristic flavan-3-ol-related fragment at *m*/*z* 289.1 in APCI-MS/MS analysis. Because the ionization and fragmentation conditions differed from those used in the present study, this literature evidence was used only as supporting evidence for the tentative annotation.

### 2.4. Molecular Docking

The docking protocol was validated by redocking the co-crystallized ligands into their respective binding sites. The redocking procedure yielded RMSD values of 0.86 Å for α-D-glucopyranose in 3A4A and 1.10 Å for acarbose in 3TOP. These values indicated that the experimentally determined ligand-binding poses were successfully reproduced by the docking protocol.

Molecular docking was performed using the catalytic domains of *S. cerevisiae* α-glucosidase (PDB ID: 3A4A) and human maltase-glucoamylase (PDB ID: 3TOP) to evaluate the binding characteristics of the putatively identified metabolites. Validation of the docking protocol showed that redocking of the native ligands produced RMSD values of 0.86 Å for α-D-glucopyranose in 3A4A and 1.1 Å for α-acarbose in 3TOP, indicating satisfactory reproduction of the crystallographic binding poses. All annotated metabolites were successfully accommodated within the catalytic pockets of both enzymes and were found to interact with residues involved in ligand recognition (Table 5).

**Figure 3:**
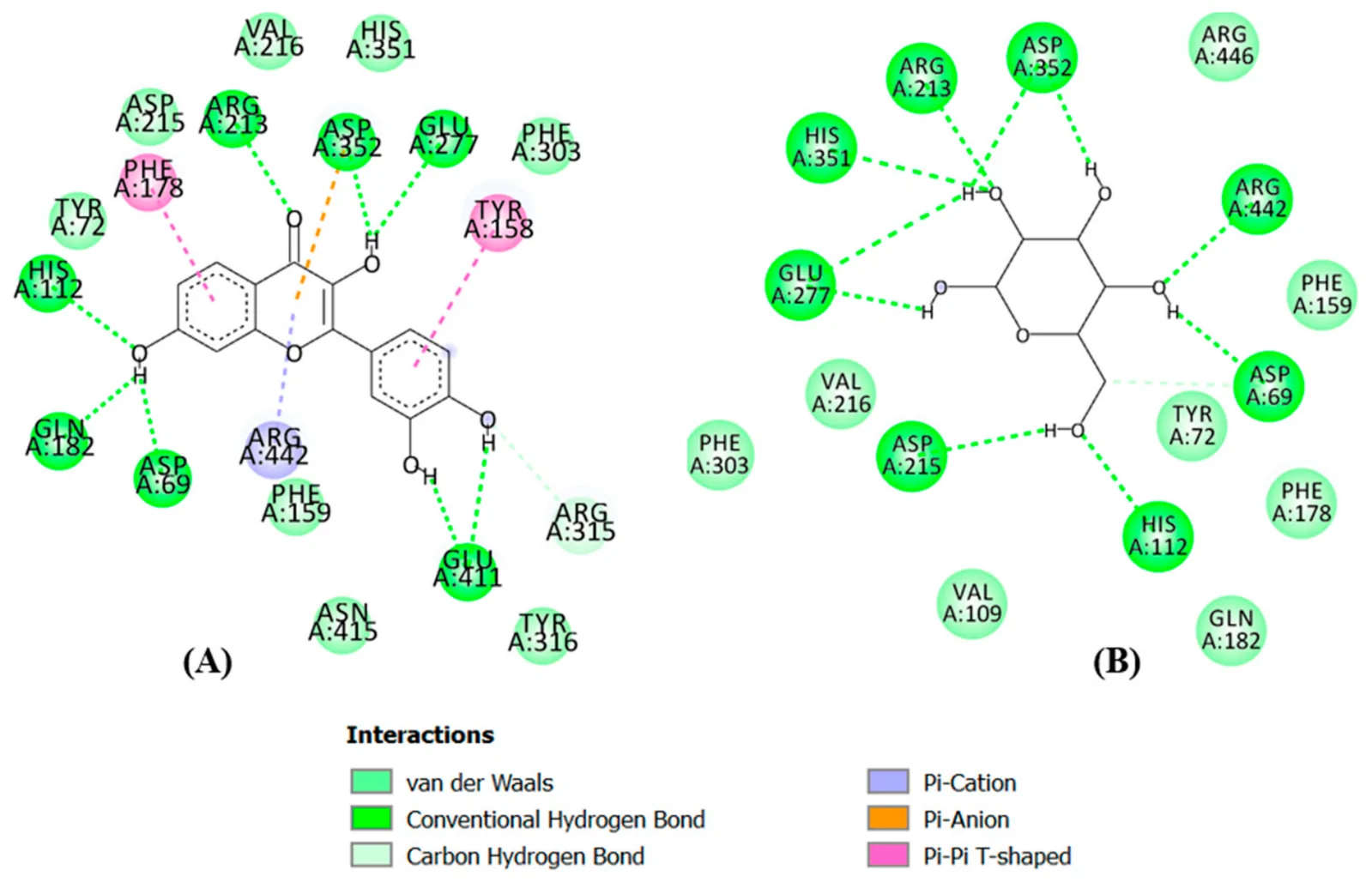
The 2D interactions of (**A**) Fisetin and (**B**) a-D-Glucopyranose. Dashed lines indicate specific interactions between the ligand and amino acid residues.

For the 3A4A model, the predicted binding energies of the annotated metabolites ranged from +639.14 to −6.87 kcal/mol (Table 5). The native ligand, α-D-glucopyranose, exhibited the lowest binding energy (−7.04 kcal/mol), whereas fisetin showed the strongest affinity among the investigated metabolites (−6.87 kcal/mol). Fisetin established conventional hydrogen bonds with Asp69, Gln182, His112, Arg213, Asp352, Glu277, and Glu411, together with π-anion and π-cation interactions involving Arg442 and Tyr158 (Figure 3A). In comparison, α-D-glucopyranose formed an extensive hydrogen-bonding network with Asp69, Asp215, Asp352, Arg213, Arg442, Glu277, His112, and His351 (Figure 3B). Scopoletin and 3,4-dihydroxybenzaldehyde displayed intermediate binding affinities, whereas 4-methoxycinnamic acid and lindetannin showed comparatively weaker docking scores (Table 5). Lindetannin showed an anomalously high positive docking score (+639.14 kcal/mol), reflecting unfavorable intermolecular interactions; therefore, it was excluded from comparative binding-affinity analysis.

**Figure 4:**
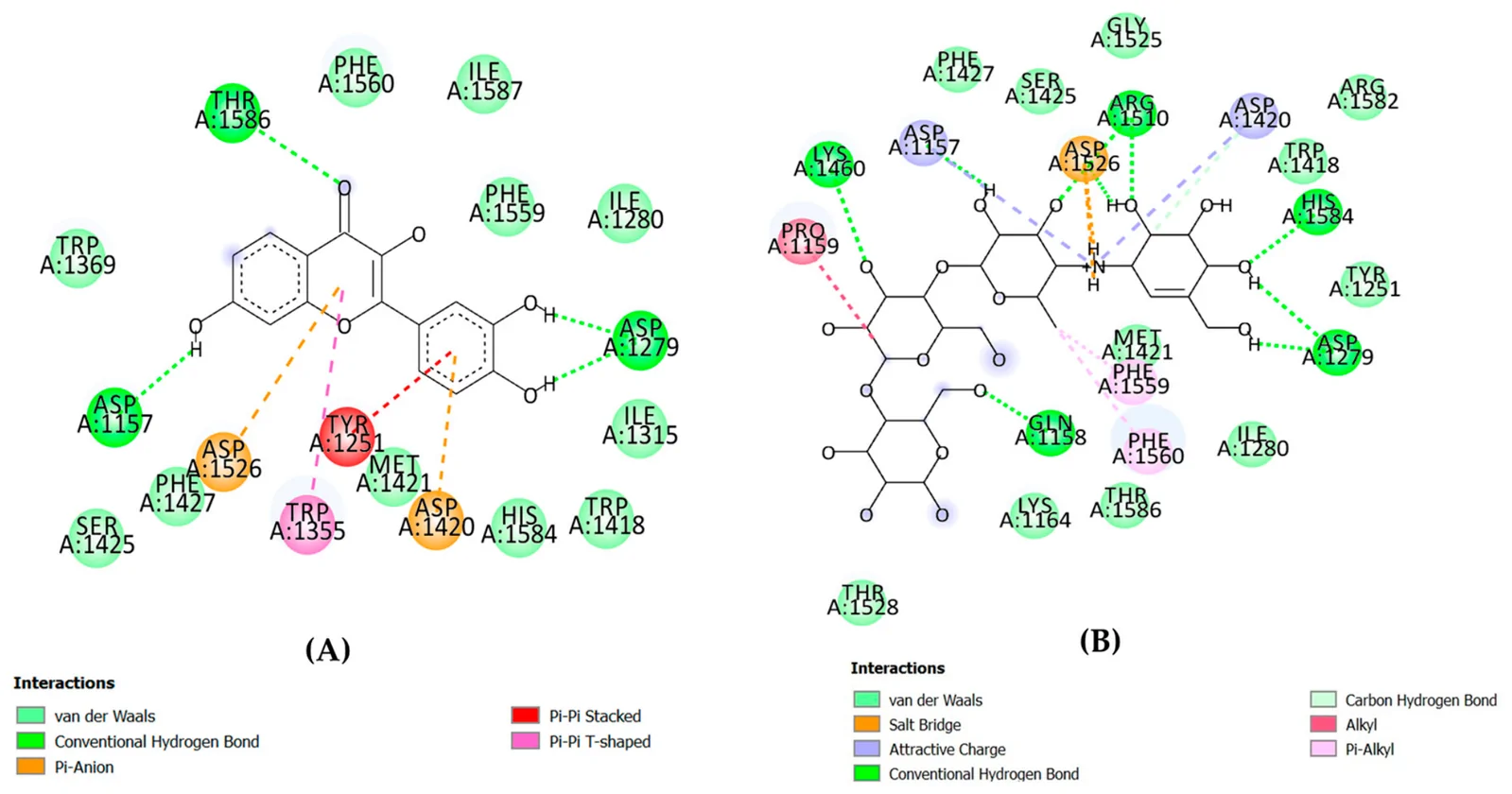
The 2D interactions of (**A**) Fisetin and (**B**) α-Acarbose. Dashed lines indicate specific interactions between the ligand and amino acid residues.

A comparable binding pattern was observed for the human maltase-glucoamylase model (3TOP). The predicted binding energies ranged from −1.61 to −7.11 kcal/mol, while the reference ligand α-acarbose exhibited the strongest affinity (−8.71 kcal/mol) (Table 5). Among the annotated metabolites, fisetin again produced the most favorable binding energy (−7.11 kcal/mol). The docked complex formed conventional hydrogen bonds with Thr1586, Asp1157, and Asp1279, as well as π-alkyl and π–π T-shaped interactions with Met1421, Tyr1251, and Phe1560 (Figure 4A). By comparison, α-acarbose interacted with His1584, Arg1510, Gln1158, and Asp1279 through an extensive hydrogen-bonding network (Figure 4B). Scopoletin and 3,4-dihydroxybenzaldehyde exhibited intermediate binding affinities, whereas 4-methoxycinnamic acid and lindetannin showed lower predicted binding energies. Lindetannin formed hydrogen bonds with Gln1561, Thr1369, Asp1326, Asp1157, Lys1164, Lys1460, and Gln1158 despite its comparatively weak docking score (Table 5).

### 2.5. Metabolomics-Guided Isolation and Structural Characterization of Scopoletin

**Figure 5:**
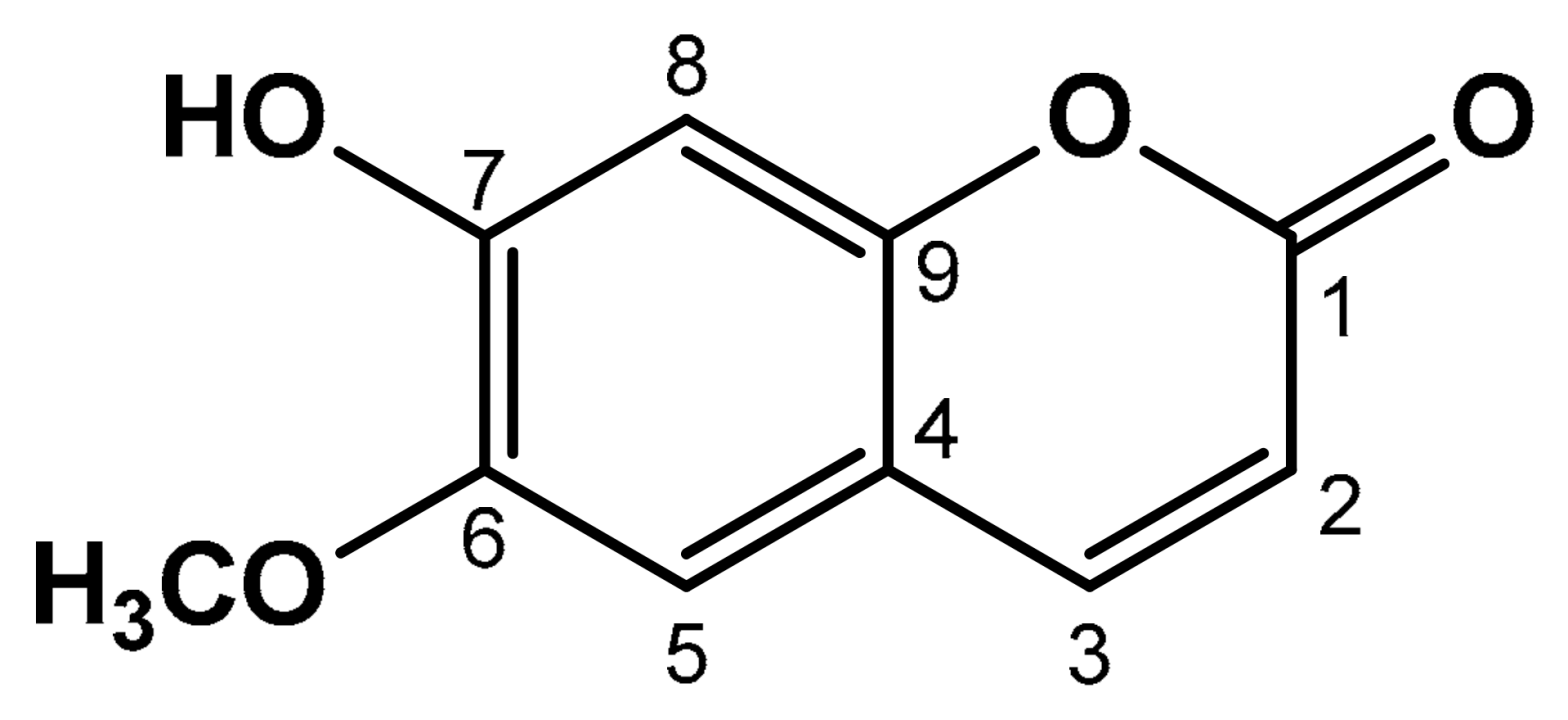
Structure of scopoletin.

Further purification of fraction F2 yielded compound **1** (52 mg), identified as scopoletin (Figure 5) by spectroscopic analyses and comparison with published data [20]. The ^1^H NMR spectrum (CDCl_3_, 700 MHz) exhibited characteristic signals at δ 7.59 (1H, d, *J* = 9.5 Hz, H-3), 6.90 (1H, s, H-5), 6.84 (1H, s, H-8), 6.26 (1H, d, *J* = 9.5 Hz, H-2), and 3.94 (3H, s, OCH_3_). The ^13^C NMR spectrum (CDCl_3_, 176 MHz) showed resonances at δ 161.7 (C-1), 149.9 (C-7), 150.4 (C-9), 144.2 (C-6), 143.5 (C-3), 113.5 (C-5), 111.6 (C-4), 107.7 (C-2), 103.4 (C-8), and 56.5 (OCH_3_).

**Figure 6:**
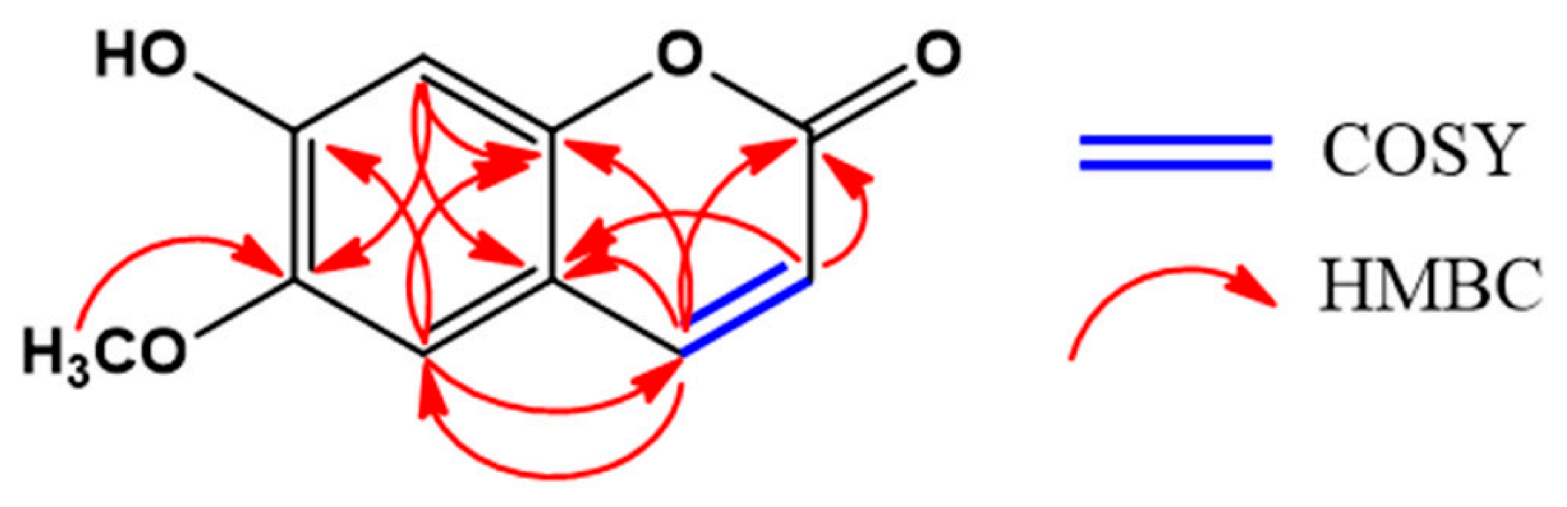
COSY and HMBC correlations of scopoletin.

The COSY correlations (Figure 6) indicated a cross-peak between H-2 (δ 6.26) and H-3 (δ 7.59). The HMBC correlations (Figure 6) from H-2 and H-3 to C-1 (δ 161.7), C-4 (δ 111.6), and C-9 (δ 150.4) indicated the presence of a lactone ring. The methoxy protons (δ 3.85) showed an HMBC correlation with C-6 (δ 144.2). In addition, HMBC correlations from H-8 (δ 6.84) to C-4, C-6, C-7 (δ 149.9) and C-9 (δ 150.4), as well as from H-5 (δ 6.90) to C-7 and C-9, indicated a substituted aromatic ring. Furthermore, HMBC correlations from H-3 to C-5 (δ 113.5) and from H-5 to C-3 (δ 143.5) supported a coumarin (benzopyran-2-one) skeleton.

## 3. Discussion

The clear differences in AGI activity among the solvent-gradient fractions show that the type of solvent mixture, not just its polarity, affects the enrichment of bioactive compounds from *P. pinnata* stem bark. Mixtures of ethyl acetate and methanol in intermediate ratios consistently showed stronger inhibitory effects than either solvent alone. This suggests that their combined properties create a better environment for extracting metabolites linked to α-glucosidase inhibition. Ethyl acetate mainly extracts semipolar compounds, while methanol is better at dissolving more polar phytochemicals, especially phenolics. Using both solvents together can broaden the range of metabolites extracted and improve selectivity, enabling more efficient recovery of compounds with different polarities than using a single solvent. This matches our earlier LC–HRMS analysis of the ethyl acetate extract from *P. pinnata* stem bark, which found phenolic acids, flavonoids, coumarins, steroids, and alkaloids, with cianidanol and fisetin as key AGI-related flavonoids [10]. It also aligns with earlier studies showing that mixed-solvent systems can improve solubilization, mass transfer, and metabolite recovery due to their combined properties rather than just polarity [21]. All fractions had lower IC_50_ values than acarbose, even though they are complex mixtures rather than pure compounds. This suggests their inhibitory effects may come from several enriched metabolites working together. The higher activity in the intermediate ethyl acetate–methanol fractions further supports the link between solvent composition and the extraction of AGI-related metabolites. However, these results do not show exactly how the solvents interact with the metabolites, so they should be seen as evidence of a connection between extraction composition and AGI activity.

The OPLS model showed a significant association between the LC–HRMS metabolic profiles and AGI activity, as indicated by a cumulative R^2^*_Y_* of 0.601, a Q^2^ of 0.435, and a significant CV-ANOVA result (*p* = 0.0084). The permutation test yielded regression intercepts of 0.273 for R^2^ and −0.336 for Q^2^. Both intercepts were below the recommended thresholds of 0.40 for R^2^ and 0.05 for Q^2^, respectively. The negative Q^2^ intercept further indicated that the permuted models had lower predictive performance than the original model, providing additional support for the validity and robustness of the OPLS model and suggesting a low risk of overfitting [13,22,23].

Clear separation of the solvent-gradient fractions in the score plot further demonstrated that solvent composition strongly influenced the metabolic profile. The 75% and 50% ethyl acetate fractions clustered closely, indicating similar chemical compositions, whereas the 100% ethyl acetate fraction formed a distinct cluster, reflecting a more restricted metabolite profile. The superior AGI activity of the 75% ethyl acetate fraction, therefore, most likely resulted from selective enrichment of bioactive constituents rather than from a larger number of detected features.

The OPLS analysis showed that a subset of LC–HRMS features contributed to the modeled AGI activity, whereas most variables were near the center of the loading plot and made relatively minor contributions to the model. Features with VIP values >1 were therefore prioritized for further investigation. The CV-ANOVA result was statistically significant (*p* = 0.0084), and the permutation test further supported the validity of the OPLS model.

Of the 14 prioritized features, five were tentatively annotated based on accurate-mass measurements and available MS/MS data. Although VIP values indicate the strength of a feature’s association with the modeled AGI activity, they do not establish its chemical identity or demonstrate a direct contribution to the observed bioactivity. The remaining nine features were retained as prioritized LC–HRMS features but could not be reliably annotated because the available MS/MS evidence and database matches were insufficient to support specific metabolite assignments.

These five metabolites represent structurally diverse classes, including flavonols, phenolic acids, phenolic aldehydes, coumarins, and condensed tannins. LC–HRMS/MS spectra and fragmentation data supporting these tentative annotations are presented in the Supplementary Materials (Section S2). To our knowledge, none of these annotated metabolites has previously been reported from *P. pinnata* stem bark, highlighting the chemical information obtained through the metabolomics-guided workflow. Several of the tentatively annotated compounds, however, have been associated with α-glucosidase inhibition or antidiabetic activity in other experimental systems. Fisetin has been reported as a potent α-glucosidase inhibitor in *Cotinus coggygria* [24]. 3,4-Dihydroxybenzaldehyde has also been shown to inhibit α-glucosidase in a structure–activity study of benzaldehyde derivatives [25], although it was not the compound isolated as the active constituent from *Periploca sepium* Bunge. 4-Methoxycinnamic acid has been isolated from *Crateva religiosa*, whereas the hypoglycemic activity reported in that study was evaluated primarily for the plant extract rather than for the isolated compound individually [26]. Evidence for the biological activity of lindetannin remains limited; therefore, no specific α-glucosidase inhibitory effect is assigned to this compound on the basis of the present annotation alone. Future studies may further clarify the individual contributions of these metabolites through quantitative analysis and direct evaluation of bioactivity.

PDB ID 3A4A was selected for the yeast α-glucosidase docking analysis because it represents an experimentally determined crystal structure of *Saccharomyces cerevisiae* isomaltase with a defined ligand-binding site [27]. The co-crystallized ligand provides structural information on ligand recognition and the arrangement of residues within the binding site. Therefore, this structure was considered an appropriate structural template for evaluating the predicted interactions of the tentatively annotated metabolites.

The strong predicted binding of fisetin may be due to its molecular structure. Fisetin, a flavonol, has a rigid, almost flat aromatic core and four hydroxyl groups that can both donate and accept hydrogen bonds. This allows the molecule to form several stabilizing interactions and remain properly positioned in the catalytic pocket. Its extended aromatic system may also help form additional noncovalent interactions within the binding pocket. In contrast, scopoletin and 3,4-dihydroxybenzaldehyde have fewer functional groups that can simultaneously interact with catalytic residues, consistent with their moderate binding affinities. The smaller size of 4-methoxycinnamic acid also limits the number of productive contacts it can make in the active site. These differences may explain the variations in docking scores seen in this study and suggest how ligands and proteins interact. However, they should not be taken as direct evidence of the strength of the inhibition or binding.

The docking results for lindetannin show how important it is for a ligand to fit well with its binding site. Even though lindetannin made several hydrogen bonds and other non-covalent interactions, its predicted binding score was still relatively low. This suggests that simply having more intermolecular contacts does not guarantee a stronger binding score. Factors such as ligand orientation, steric fit, molecular shape, and the arrangement of interacting groups can also affect the calculated interaction energy. Molecular docking provides only a static view of ligand–protein interactions and does not fully capture receptor flexibility, solvent effects, or binding kinetics. Because of this, docking results should be seen as supporting evidence for choosing metabolites, not as direct proof of enzyme inhibition.

Metabolomics-guided isolation of the most active fraction yielded compound **1**, which was identified as scopoletin by NMR analysis. To the best of our knowledge, this represents the first report of scopoletin isolated from *P. pinnata* stem bark. Although scopoletin was prioritized by the OPLS model and detected in the fraction showing the strongest AGI activity, the isolated compound exhibited only weak inhibitory activity (IC_50_ > 200 µg/mL), whereas the corresponding parent fraction showed substantially stronger activity (IC_50_ = 5.53 µg/mL). This difference indicates that the activity of the parent fraction cannot be attributed to scopoletin alone and may reflect the combined contribution of multiple constituents. A synergistic interaction is one possible explanation, but this hypothesis requires direct experimental testing and should not be inferred solely from the fraction’s higher activity [28]. Experimental evidence from glyceollin and luteolin demonstrates that synergistic inhibition is compound- and ratio-dependent. Their combination produced combination index values below 1 across several ratios, with the lowest value of 0.64244 at a 3:7 ratio, whereas combinations involving glyceollin with genistein or daidzein did not show synergistic effects [29]. Thus, the greater activity of a plant fraction compared with an isolated constituent should not by itself be interpreted as evidence of synergy.

Similarly, epicatechin and citric acid were suggested to interact well in an insulin receptor docking model. However, these compounds were not tested together in experiments, so their interaction was not proven to be synergistic [30]. A similar idea was proposed for the fractionation of *Euphorbia dendroides* latex, in which synergism was thought to account for the loss of antiviral activity during fractionation, but this was not demonstrated experimentally [31]. These cases highlight that synergistic interactions need to be shown through experiments, not just inferred from activity at the fraction level.

The marked difference in activity between the isolated compound and the parent fraction suggests that the inhibitory effect of *P. pinnata* stem bark may result from the combined contribution of several metabolites rather than from a single dominant constituent. In this regard, the OPLS model should be regarded primarily as a tool for prioritizing metabolites associated with bioactivity, rather than for identifying the principal active compound. Similar observations have been reported in metabolomics-guided studies of natural products, in which metabolites that show strong correlations with biological activity do not necessarily retain comparable potency when evaluated individually.

Previous studies have reported varying levels of AGI activity for scopoletin across experimental systems and plant sources. Tan et al. [32] reported strong AGI for scopoletin isolated from *Paederia foetida*, with an IC_50_ of 0.057 µM. In contrast, Zhang et al. [33] reported weak AGI activity of the methanolic extract of *Heracleum dissectum*, despite significant antidiabetic effects in vivo. These differences indicate that the biological activity observed in plant extracts cannot necessarily be attributed to scopoletin alone and may depend on compound concentration, matrix composition, or other constituents. In the present study, the weak activity of isolated scopoletin compared with the corresponding parent fraction suggests that scopoletin is unlikely to be the principal contributor to the α-glucosidase inhibitory activity of *P. pinnata* stem bark.

## 4. Materials and Methods

### 4.1. Plant Materials

*Pometia pinnata* J.R. Forst. & G. Forst. (Sapindaceae), commonly known as Matoa, was collected on 10 January 2025, from an authenticated living collection at the Provincial Botanical Garden, B.J. Habibie Science and Technology Area, National Research and Innovation Agency (BRIN), Banten, Indonesia (6°21′18.52″ S, 106°40′0.39″ E). Botanical identification was performed at Herbarium Bogoriense, Directorate of Scientific Collection Management, National Research and Innovation Agency (BRIN), and the taxonomic identity was confirmed by comparison with the Herbarium Bogoriense reference specimen (No. BO-1349401).

### 4.2. Chemicals and Reagents

Analytical-grade reagents and solvents were purchased from Merck (Darmstadt Germany), including TLC silica gel 60 F254, silica gel 60 GF254, bovine serum albumin, potassium persulfate, sodium carbonate, dimethyl sulfoxide, dichloromethane, chloroform, sodium hydroxide, and methanol. Phosphate buffer (pH 7.0), *p*-nitrophenyl-α-D-glucopyranoside (*p*NPG), acarbose (Sigma-Aldrich, St. Louis, MO, USA), and α-glucosidase from *Saccharomyces cerevisiae* (Wako Pure Chemicals Corporation, Osaka, Japan) were used for the enzymatic assay. LC-MS-grade solvents were obtained from Fisher Scientific (Fair Lawn, NJ, USA). Multivariate statistical analyses were conducted using MZmine 2.53 and SIMCA-P+ 14. Molecular docking studies were performed using Chimera, Avogadro 1.2.0, ORCA, AutoDockTools 1.5.6, and Discovery Studio Visualizer v17.2.0.

### 4.3. Extraction, Solvent Fractionation, and AGI Assay

The collected stem bark of *P. pinnata* was washed, dried in a forced-air oven at 45 °C for 48 h, and ground into a fine powder. The powdered material was sequentially extracted with *n*-hexane followed by ethyl acetate at a material-to-solvent ratio of 1:5 (*w*/*v*). This extraction yielded 41.67 g of *n*-hexane extract (0.20%, *w*/*w*) and 369.16 g of ethyl acetate extract (1.76%, *w*/*w*). Based on its previously reported AGI activity, the ethyl acetate extract was selected for subsequent metabolomic profiling [10].

To generate samples with varied metabolite profiles for metabolite–bioactivity correlation analysis, the ethyl acetate extract was fractionated using ethyl acetate–methanol mixtures of increasing polarity. Five solvent mixtures were employed: 100% ethyl acetate (EA1–EA6), 75% ethyl acetate in methanol (75%EA1–75%EA6), 50% ethyl acetate in methanol (50%EA1–50%EA6), 25% ethyl acetate in methanol (25%EA1–25%EA6), and 100% methanol (M1–M6). A 300 mg portion of the ethyl acetate extract was dissolved in the corresponding solvent mixture, sonicated at room temperature and then centrifuged. For each solvent mixture, six independent replicates were obtained from different portions of the same ethyl acetate extract. These replicates served as independent samples for the OPLS analysis. We prepared six separate fractions from each solvent mixture, for a total of 30 fractions. The supernatants were collected and allowed to evaporate at room temperature before LC–HRMS analysis and AGI activity testing.

The AGI activity of the fractions was measured using the *p*-nitrophenyl-α-D-glucopyranoside (*p*NPG) substrate assay, following a previously reported method [10]. The assay tested concentrations ranging from 3.125 to 200 μg/mL to assess concentration-dependent inhibition and estimate IC_50_ values. α-Glucosidase from *S. cerevisiae* was the enzyme used, and acarbose was the reference inhibitor.

Absorbance was measured at 405 nm using a microplate reader. Four measurement conditions were included to account for background and sample-derived absorbance: *A*1 (reagent blank), containing the assay reagents without the test fraction and with inactivated enzyme; *A*2 (enzyme control), containing all assay components except the test fraction, with active enzyme; *A*3 (sample blank), containing the test fraction and assay reagents with inactivated enzyme; and *A*4 (sample reaction), containing the test fraction and all assay components, including active enzyme. The percentage inhibition was calculated using the following equation (Equation (1)):

(1) $$\mathrm{Inhibition}\;\left(\%\right)=\left[1-\frac{\left(A4-A3\right)}{\left(A2-A1\right)}\right]\times 100\%$$

Here, (*A*2 − *A*1) represents the net absorbance of the uninhibited enzyme reaction after correction for assay background, whereas (*A*4 − *A*3) represents the corresponding enzyme reaction signal in the presence of the test fraction after correction for sample-derived absorbance. Thus, the contribution of the test fraction to the measured absorbance was accounted for before calculating enzyme inhibition. All measurements were performed in triplicate, and IC_50_ values were calculated from the logarithmic concentration–inhibition curves.

### 4.4. LC-HRMS Analysis

Sample preparation, chromatographic separation, mass spectrometric acquisition, and metabolite annotation were performed as previously reported for LC–HRMS methods [10,34]. Briefly, 50 mg of each dried fraction was dissolved in 1 mL of LC–MS-grade acetonitrile, vortex-mixed for 30 s, sonicated for 30 min at room temperature, centrifuged at 1400× *g* for 5 min, and filtered through a 0.22 μm PTFE membrane prior to analysis. Metabolite profiling was carried out on a Thermo Scientific Vanquish UHPLC coupled with a Q Exactive Hybrid Quadrupole-Orbitrap mass spectrometer operating in positive electrospray ionization mode using full-scan MS and data-dependent MS/MS (dd-MS^2^). Xcalibur™ 4.4 software was used for instrument control and data acquisition, whereas Compound Discoverer^®^ was employed for feature extraction and preliminary metabolite annotation. Putative metabolite identification was achieved by matching accurate mass and MS/MS fragmentation spectra to the mzCloud™ and ChemSpider™ databases, with a mass tolerance of ±5 ppm.

### 4.5. LC-HRMS Data Processing and Multivariate Analysis

Raw LC–HRMS data were processed in MZmine 2.53 using a feature-based untargeted metabolomics workflow that included baseline correction, mass detection, chromatogram construction, peak deconvolution, isotopic grouping, duplicate peak removal, feature alignment, normalization, and gap filling. Baseline correction was carried out using an asymmetry factor of 0.5–0.7 and a smoothing factor of 100,000. Mass detection was performed in centroid mode with a noise threshold of 2500–3000 counts. Chromatograms were generated using a minimum time span of 0.2 min, a minimum peak height of 2500, and *m*/*z* tolerances of 1 *m*/*z* and 2500 ppm. Peaks were smoothed using an 11-point algorithm before deconvolution with a baseline cut-off of 2500. Isotopic features were grouped using a retention time tolerance of 0.2–0.5 min, and duplicate peaks were removed using the same *m*/*z* tolerance criteria. To reduce analytical variation among injections, peak intensities were normalized to the total ion signal. Feature alignment was performed using *m*/*z* and retention time weighting factors of 85 and 15, respectively, and required identical charge states and feature IDs. Missing signals were subsequently recovered via gap filling using the same *m*/*z* and retention-time tolerances. The processed feature matrix, containing feature IDs, retention times, *m*/*z* values, and normalized peak areas, was exported as a CSV file for subsequent multivariate analysis.

The metabolomics dataset comprised LC–HRMS features defined by retention time and *m*/*z* values from 30 fractions, together with the corresponding AGI activities expressed as 1/IC_50_ AGI. Each feature represented the measured signal intensity at a specific retention time–*m*/*z* pair, with zero values indicating that the feature was not detected in a given sample. Before OPLS analysis, all variables were unit-variance (UV) scaled to ensure equal feature weighting and to minimize the influence of variables with large variances, thereby improving comparability across the dataset [13].

The scaled dataset was subsequently analyzed by OPLS to examine the relationship between metabolite profiles and AGI activity. Model quality was evaluated using cumulative R^2^*_Y_* and Q^2^ values; a difference of ≤0.3 between them was considered indicative of a robust model with minimal risk of overfitting. Metabolites contributing to model discrimination were identified using VIP scores and loading scatterplots; variables with VIP values > 1 were considered important contributors [35]. Model significance was further verified by cross-validated analysis of variance (CV-ANOVA), with *p* ≤ 0.05 considered statistically significant.

### 4.6. Molecular Docking Simulation Methods

Molecular docking was performed to investigate the potential binding interactions of the tentatively annotated metabolites with *Saccharomyces cerevisiae* α-glucosidase and human maltase-glucoamylase. PDB IDs 3A4A and 3TOP were selected because they contain experimentally determined crystal structures with defined ligand-binding sites and co-crystallized ligands, enabling subsequent validation of the docking protocol by redocking. PDB ID 3A4A corresponds to the ligand-bound crystal structure of *S. cerevisiae* isomaltase [27], an enzyme belonging to the α-glucosidase family, determined by X-ray crystallography at 1.60 Å resolution with α-D-glucopyranose in the active site. PDB ID 3TOP represents the C-terminal subunit of human maltase-glucoamylase in complex with the inhibitor acarbose, determined at 2.88 Å resolution. Accordingly, 3A4A and 3TOP were used as structural models for the yeast α-glucosidase and human intestinal maltase-glucoamylase systems, respectively.

The protein structures were prepared using UCSF Chimera by removing co-crystallized ligands and other nonessential molecules, while retaining the native protein structures for docking [36]. The investigated metabolites were retrieved from PubChem and converted into three-dimensional structures using Avogadro 1.2.0. Ligand geometries were subsequently optimized using density functional theory (DFT) at the B3LYP/3-21G level in ORCA to obtain optimized ligand conformations prior to docking [36].

Docking calculations were performed using AutoDockTools 1.5.6 with a 30 × 30 × 30 Å^3^ grid box centered on the catalytic site of each enzyme [37]. Ligand conformations were sampled using the Lamarckian Genetic Algorithm, with 50 independent docking runs performed for each ligand–protein complex. Docking protocol validation was performed by removing the corresponding crystallographic ligand from each binding site and subsequently redocking it into its original binding site [38]. The resulting docking poses were compared with the experimentally determined ligand conformations using root-mean-square deviation (RMSD). The binding conformations and ligand–protein interactions were subsequently analyzed and visualized using Discovery Studio Visualizer v17.2.0 [39].

### 4.7. Isolation and NMR Identification

The isolation process followed the OPLS model to highlight metabolite features associated with AGI activity. To start, 90 g of the ethyl acetate extract was dissolved in 75% ethyl acetate in methanol and separated by vacuum silica gel column chromatography using a stepwise gradient of *n*-hexane and ethyl acetate (7:3, 5:5, and 4:6, *v*/*v*), followed by 100% methanol. This process produced three main fractions (F1–F3), monitored by thin-layer chromatography (TLC). The OPLS loading plot and VIP analysis indicated that F2 contained metabolite features associated with AGI activity, so it was chosen for further purification. Repeated silica gel column chromatography with the same solvents yielded compounds **1** (52 mg) and **2** (2.2 mg). Compound **2** and some other isolated components were not further analyzed or discussed in this study.

The structures of the isolated compounds were determined using one- and two-dimensional NMR spectroscopy, including ^1^H, ^13^C, COSY, and HMBC experiments, on a Bruker AVANCE NEO 700 MHz spectrometer (Bruker, Billerica, MA, USA). The structural assignments were made by comparing the results with previously reported spectroscopic data [20].

## 5. Conclusions

In this study, we used a metabolomics-guided approach to identify potential AGI metabolites in the stem bark of *P. pinnata*. We combined untargeted LC–HRMS, OPLS analysis, molecular docking, and metabolomics-guided isolation. Among 30 ethyl acetate–methanol gradient fractions, the 75% ethyl acetate in methanol fraction had the strongest AGI, with an IC_50_ of 5.53 μg/mL. We prioritized 14 LC–HRMS features linked to AGI and tentatively identified five: scopoletin, 3,4-dihydroxybenzaldehyde, fisetin, 4-methoxycinnamic acid, and lindetannin. To our knowledge, these compounds have not been reported before in the stem bark of *P. pinnata*. Fisetin showed the best predicted interactions with both *S. cerevisiae* α-glucosidase and human maltase-glucoamylase. We confirmed the structure of scopoletin through isolation and NMR analysis, but the isolated compound showed weak AGI (IC_50_ > 200 μg/mL). This suggests that scopoletin alone does not explain the strong activity of the parent fraction. The difference in activity between the parent fraction and isolated scopoletin indicates that other components, possibly the unannotated features, may also play a role. Overall, our work shows that an integrated metabolomics-guided strategy is useful for finding and characterizing AGI-associated metabolites in *P. pinnata* stem bark. This provides a starting point for further isolation, structure analysis, and testing of the metabolites responsible for its AGI.

## Figures and Tables

**Table 1:** α-Glucosidase inhibitory activity (IC_50_) of *Pometia pinnata* stem bark fractions.

No	Solvent System	IC_50_ (µg/mL) Mean ± SD
1	Ethyl acetate	10.68 ± 1.18 ^c^
2	75% ethyl acetate in methanol	5.53 ± 0.70 ^a^
3	50% ethyl acetate in methanol	6.95 ± 0.50 ^a^
4	25% ethyl acetate in methanol	10.77 ± 1.24 ^c^
5	Methanol	8.34 ± 0.65 ^b^

Data are presented as mean ± SD (*n* = 6). Values followed by different lowercase letters within the same column are significantly different (*p* < 0.05). Acarbose was used as the positive control (IC_50_ = 255.28 ± 31.84 μg/mL). IC_50_ = half maximal inhibitory concentration.

**Table 2:** OPLS model parameters describing the relationship between LC–HRMS metabolite profiles and α-glucosidase inhibitory activity (1/IC_50_ AGI) of *Pometia pinnata* stem bark fractions.

Model	Components	R^2^*_X_* (cum)	R^2^*_Y_* (cum)	Q^2^ (cum)	Output
OPLS	2	0.56	0.601	0.435	1/IC_50_

Abbreviations: OPLS = orthogonal partial least squares; R^2^*_X_* (cum) = cumulative explained variance of the predictor variables (*_X_*); R^2^*_Y_* (cum) = cumulative explained variance of the response variable (*_Y_*); Q^2^ (cum) = cumulative cross-validated predictive ability of the model; AGI = α-glucosidase inhibitory activity; IC_50_ = half maximal inhibitory concentration.

**Table 3:** Prioritized LC–HRMS features with VIP > 1 associated with α-glucosidase inhibitory activity in *Pometia pinnata* stem bark fractions based on OPLS analysis.

Variable ID	*m*/*z*	RT (min)	VIP Value	Loading Plot	
				*pq*[1]	*pq*[2]
662	419.3143636	16.850312	1.37643	0.0568716	0.0292776
126	403.1529958	13.575356	1.37537	0.0544374	0.0373199
352	323.2569331	15.202574	1.36218	0.0556342	0.0365509
128	395.2396957	10.647236	1.35625	0.0564092	0.0233806
291	379.2839600	15.914838	1.35991	0.0557660	0.0340665
415	433.3300664	18.295026	1.35138	0.0559655	0.0269718
297	293.2102997	12.554444	1.34885	0.0547785	0.0392932
469	275.2000397	12.554261	1.34557	0.0548043	0.0376479
187	865.1961578	5.149112	1.28274	0.0512301	0.0447070
57	287.0545823	6.043489	1.27787	0.0515753	0.0401588
72	179.0698502	8.954618	1.25985	0.0492578	0.0515401
146	139.0387204	4.527988	1.23260	0.0484499	0.0487070
4	291.0858444	4.529440	1.22536	0.0483715	0.0469945
654	193.0858294	5.786157	1.03849	0.0381632	0.0558510

Abbreviations: VIP = variable importance in projection; RT = retention time. Features are presented by their OPLS-generated variable IDs and ranked by VIP values.

**Table 4:** Tentative LC–HRMS/MS annotations of metabolites associated with α-glucosidase inhibitory activity in *Pometia pinnata* stem bark in positive ion mode.

Compound/Variable ID	Molecular Formula	Exact Mass (Da)	RT (min)	Observed Ion(s)	Fragment Ion (*m*/*z*)	Mass Error (ppm)
Scopoletin (Var ID 654)	C_10_H_8_O_4_	192.0498	5.7	[M+H]^+^	193.0498	−2.09
				[M+H−CH_3_]^+^	178.0263	
				[M+H−C_2_H_4_O_2_]^+^	133.0283	
3,4-Dihydroxybenzaldehyde (Var ID 146)	C_7_H_6_O_3_	138.038	4.5	[M+H]^+^	139.0387	−2.10
				[M+H−CO]^+^	111.0441	
				[M+H−CO−H_2_O]^+^	93.0337	
Fisetin (Var ID 57)	C_15_H_10_O_6_	286.04726	6.0	[M+H]^+^	287.0547	−1.68
				[M+H−C_8_H_6_O_3_]^+^	137.0233	
4-Methoxycinnamic acid (Var ID 72)	C_10_H_10_O_3_	178.06278	8.9	[M+H]^+^	179.0700	−1.22
				[M+H−C_3_H_4_O]^+^	123.0596	
				[M+H−C_3_H_4_O−H_2_O]^+^	105.0700	
Lindetannin (Var ID 187)	C_45_H_36_O_18_	864.19543	5.1	[M+H]^+^	865.1954	−2.32
				[M+H−C_17_H_18_O_7_]^+^	533.1073	
				[M+H−C_38_H_30_O_16_]^+^	123.0440	
				[M+H−H_2_O−C_30_H_24_O_11_]^+^	287.0546	
				[M+H−H_2_O−C_30_H_24_O_11_−CH_2_O]^+^	257.0441	

**Table 5:** Binding affinities and hydrogen-bond interactions of putatively identified metabolites with α-glucosidase (PDB ID: 3A4A) and human maltase-glucoamylase (PDB ID: 3TOP).

Compound	*S. cerevisiae* α-Glucosidase (PDB ID: 3A4A)		Human Maltase-Glucoamylase (PDB ID: 3TOP)	
	Binding Energy (kcal/mol)	H-Bond	Binding Energy (kcal/mol)	H-Bond
Scopoletin	−4.6	Asp215, Arg213	−5.70	Asp1279, His1584
3,4-Dihydroxybenzal-dehyde	−5.74	His112, Asp352, Arg213	−5.07	Arg1510, Asp1279, His1584
Fisetin	−6.87	Asp69, Gln182, His112, Arg213, Asp352, Glu277, Glu411	−7.11	Thr1586, Asp1157, Asp1279
4-Methoxycinnamic acid	−2.25	Gln279	−3.73	Asp1157
Lindetannin	+639.14	-	−1.61	Gln1561, Thr1369, Met1421, Asp1526, Asp1157, Lys1164, Lys1460, Gln1158
a-D-Glucopyranose RMSD: 0.86	−7.04	Asp215, Glu277, His351, Arg213, Asp352, Arg442, Asp69, His112	-	-
α-Acarbose RMSD = 1.1	-	-	−8.71	His1584, Arg1510, Gln1158, Asp1279

Binding energies are expressed in kcal/mol; more negative values indicate stronger predicted binding affinity. H-bond denotes hydrogen-bond interactions between the ligand and protein residues. RMSD, Root Mean Square Deviation, was determined by native ligand redocking; RMSD values below 2.0 Å indicate acceptable docking accuracy.

## Data Availability

The original contributions presented in this study are included in this article and its Supplementary Materials. Further inquiries can be directed to the corresponding author. During the preparation of this manuscript, the authors used ChatGPT (GPT-5.6 Luna) to improve the text’s readability. The authors have thoroughly reviewed and edited AI-assisted output and take full responsibility for the content of the published article.

## Supplementary Materials

The following supporting information can be downloaded at: https://www.mdpi.com/article/10.3390/molecules31183233/s1. Table S1. Prioritized LC–HRMS features associated with α-glucosidase inhibitory activity in *Pometia pinnata* stem bark fractions and their tentative metabolite annotations.
