# Supplementary material for: Metabolomics-Based Identification of α-Glucosidase Inhibitors from Pometia pinnata Stem Bark Using LC-HRMS and Molecular Docking
Source: Molecules. 2026 Sep 13;31(18):3233. doi: 10.3390/molecules31183233 (PMC13609815; doi:10.3390/molecules31183233)

Supplementary Material

Table S1. Prioritized LC–HRMS features associated with α-glucosidase inhibitory activity in *Pometia pinnata* stem bark fractions and their tentative metabolite annotations.

Variable ID	Molecular weight	RT	VIP	Formula	Candidate	MSI	Database	Match score	Mass error	Spectrum
662	418.3143636	16.850312	1.37643	-	Unannotated	-	-	-	-	-
126	402.1529958	13.575356	1.37537	-	Unannotated	-	-	-	-	-
352	322.2569331	15.202574	1.36218	-	Unannotated	-	-	-	-	-
128	394.2396957	10.647236	1.35625	-	Unannotated	-	-	-	-	-
291	378.2839600	15.914838	1.35991	-	Unannotated	-	-	-	-	-
415	432.3300664	18.295026	1.35138	-	Unannotated	-	-	-	-	-
297	292.2102997	12.554444	1.34885	-	Unannotated	-	-	-	-	-
469	274.2000397	12.554261	1.34557	-	Unannotated	-	-	-	-	-
187	864.1961578	5.149112	1.28274	$C_{45}H_{36}O_{18}$	Lindetannin	Level 2	mzCloud ChemSpider	94.1	-2.6	DDA-MS/MS; Supplementary Material S2 E.1-4
57	286.0545823	6.043489	1.27787	$C_{15}H_{10}O_6$	Fisetin	Level 2	mzCloud ChemSpider	97.6	-3.06	DDA- MS/MS; Supplementary Material S2 C
72	178.0698502	8.954618	1.25985	$C_{10}H_{10}O_3$	4-Methoxycinnamic acid	Level 2	mzCloud ChemSpider	87.9	-2.16	DDA- MS/MS; Supplementary Material S2 D
146	138.0387204	4.527988	1.23260	$C_7H_6O_3$	3,4-Dihydroxybenzaldehyde	Level 2	mzCloud ChemSpider	90.7	-2.43	DDA- MS/MS: Supplementary Material S2 B
4	290.0858444	4.529440	1.22536	-	Unannotated	-	-	-	-	-
654	192.0858294	5.786157	1.03849	$C_{10}H_8O_4$	Scopoletin	Level 2	mzCloud ChemSpider	99.8	-2.09	DDA- MS/MS: Supplementary Material S2 A

A dash (–) indicates that available database hits did not provide sufficient evidence to support a tentative metabolite annotation.

S2. LC–HRMS/MS Spectra and Fragmentation Pathways of Putatively Annotated Compounds

A. Scopoletin

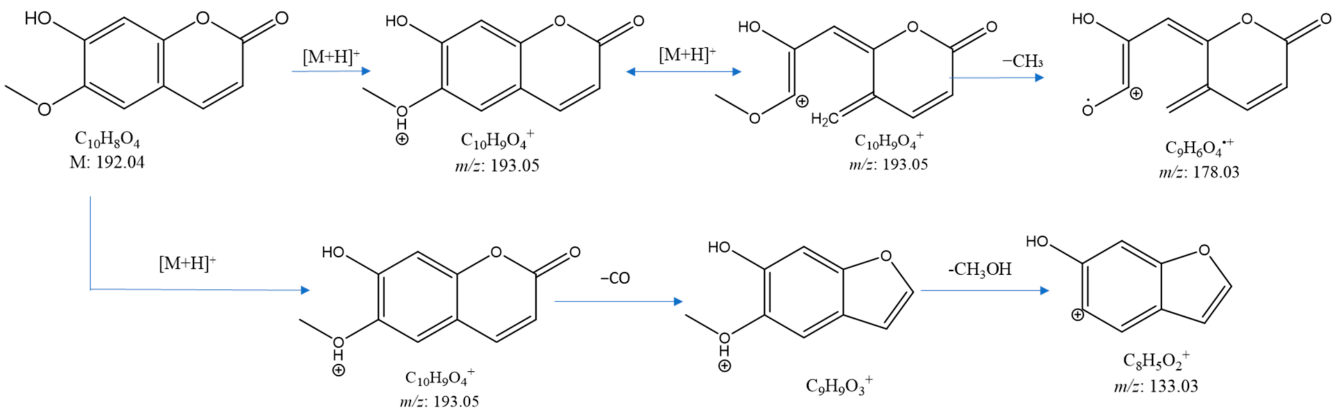

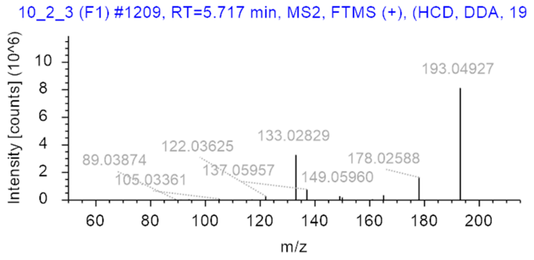

B. 3,4-Dihydroxybenzaldehyde

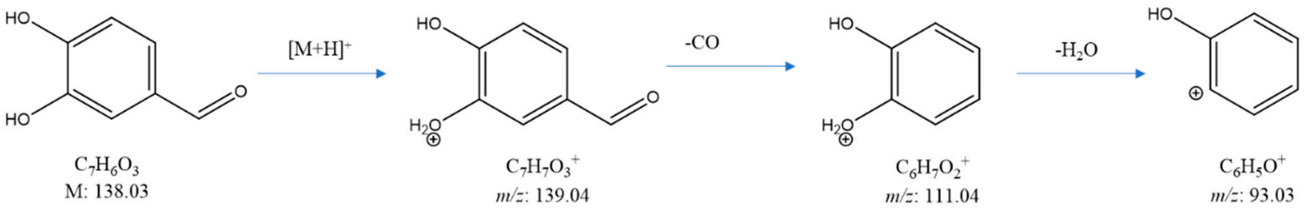

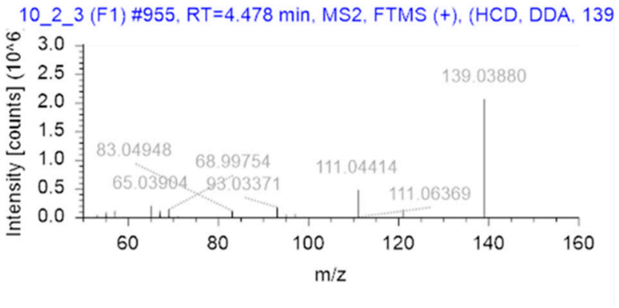

C. Fisetin

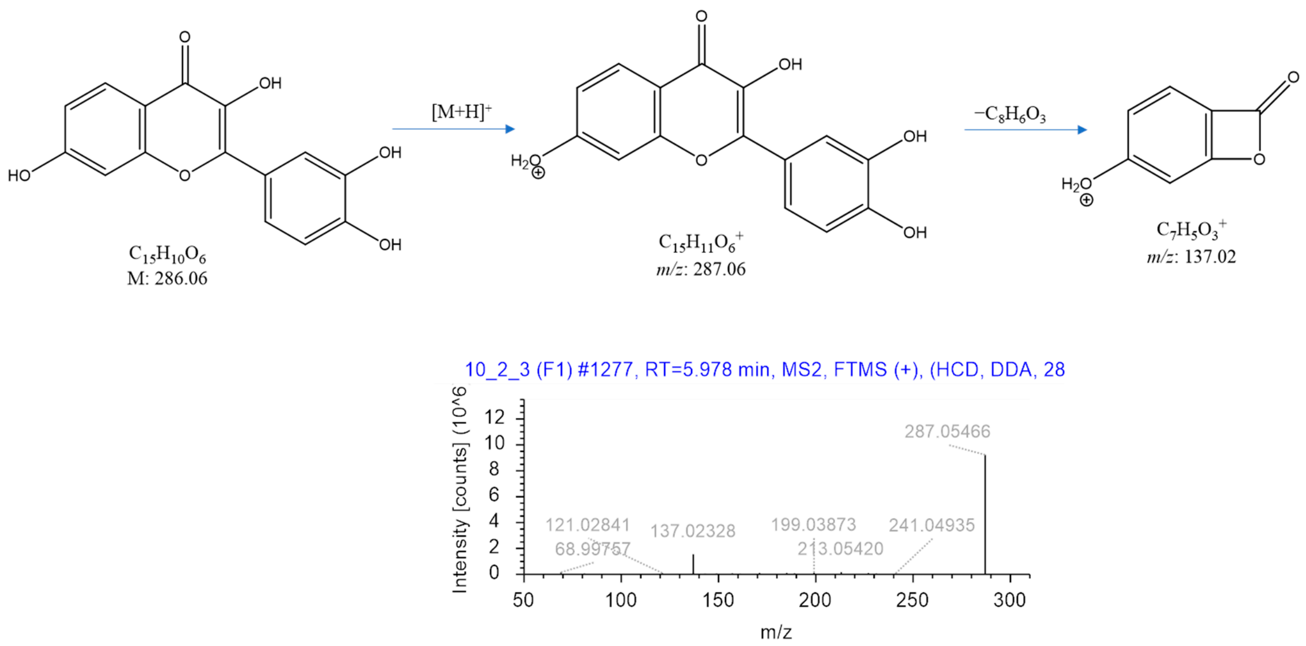

D. 4-methoxycinnamic acid

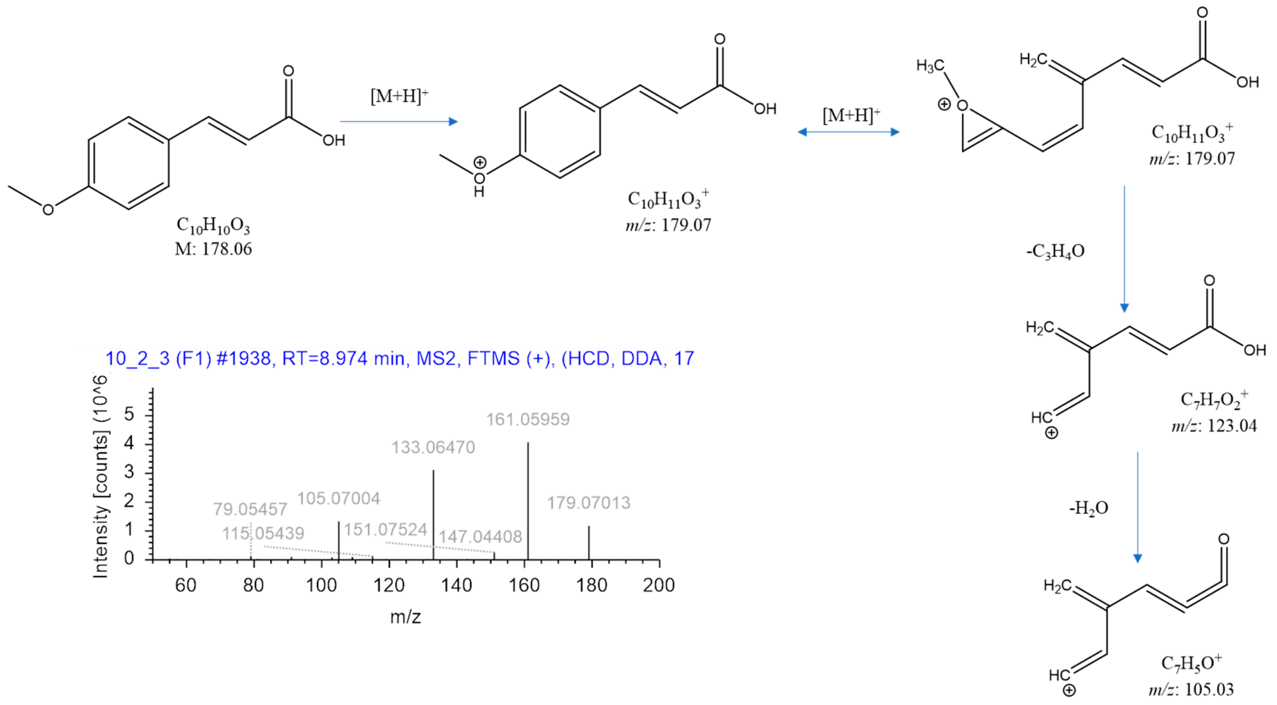

E. Fragmentation Pathway of Lindetannin leading to product ions at *m/z* 533, 123, 257, and 287.

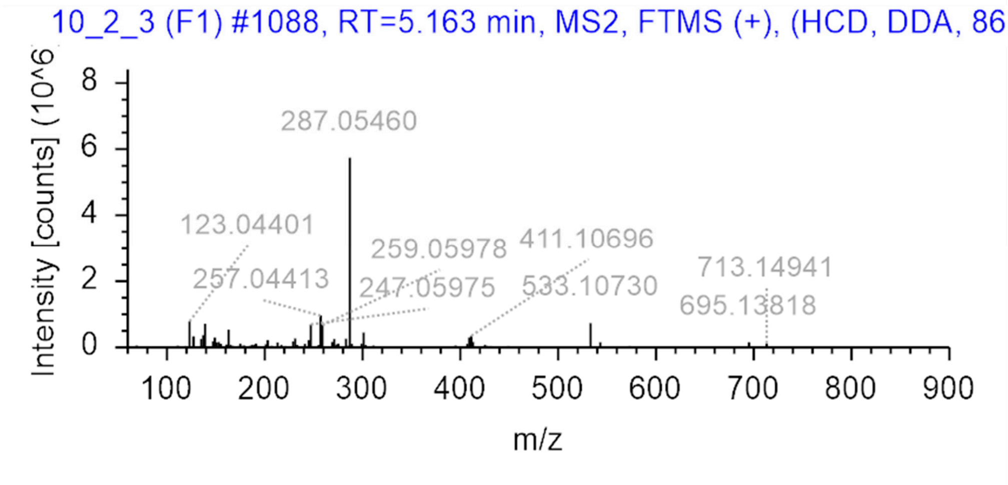

1. Fragmentation Pathway of Lindetannin leading to product ion at *m/z* 533

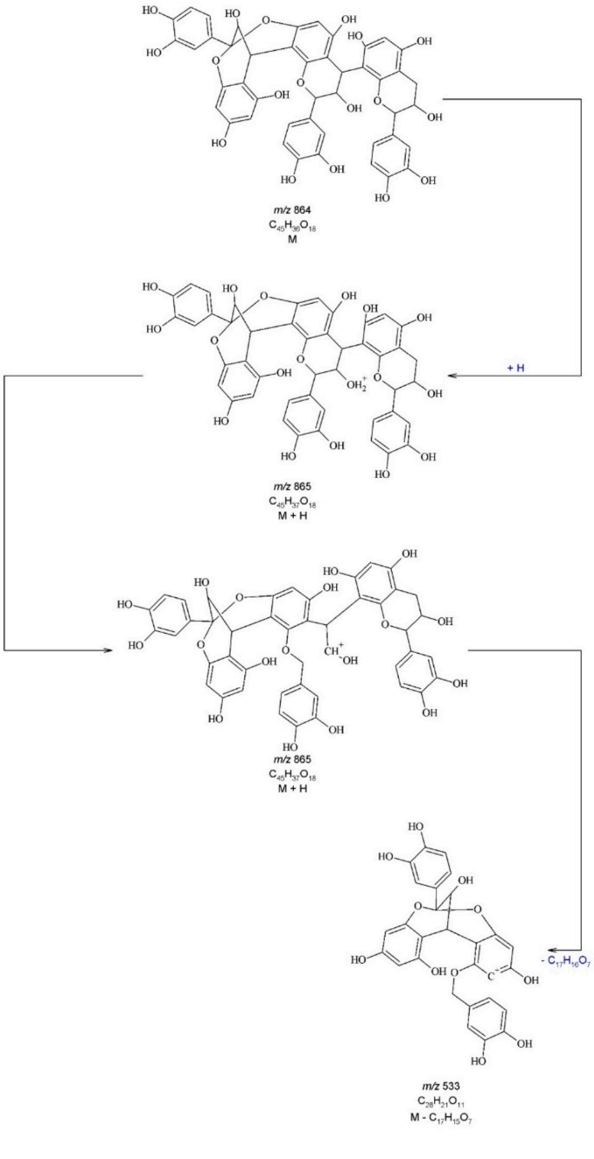

2. Fragmentation Pathway of Lindetannin leading to product ion at m/z 123

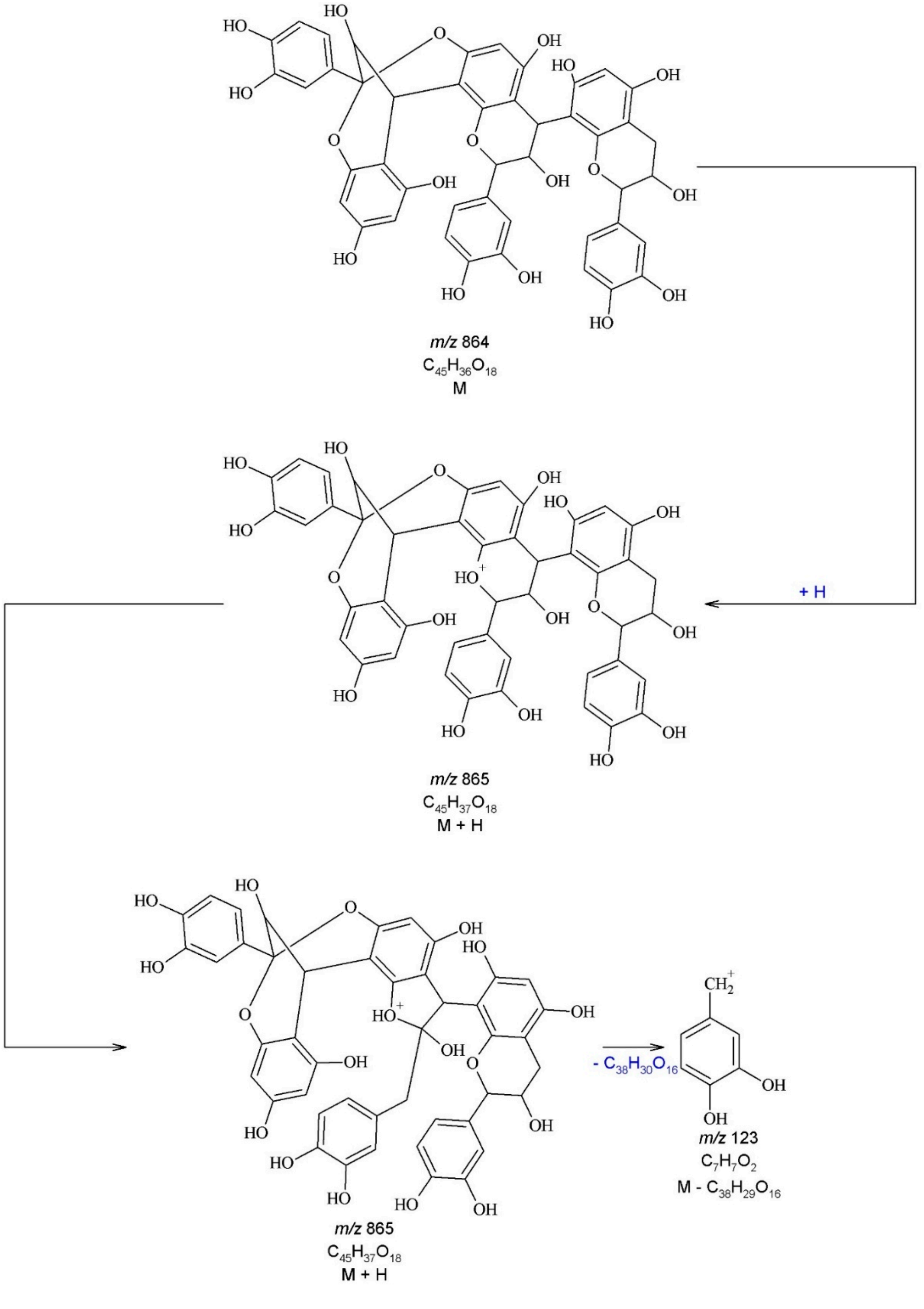

3. Fragmentation Pathway of Lindetannin leading to product ions at m/z 257

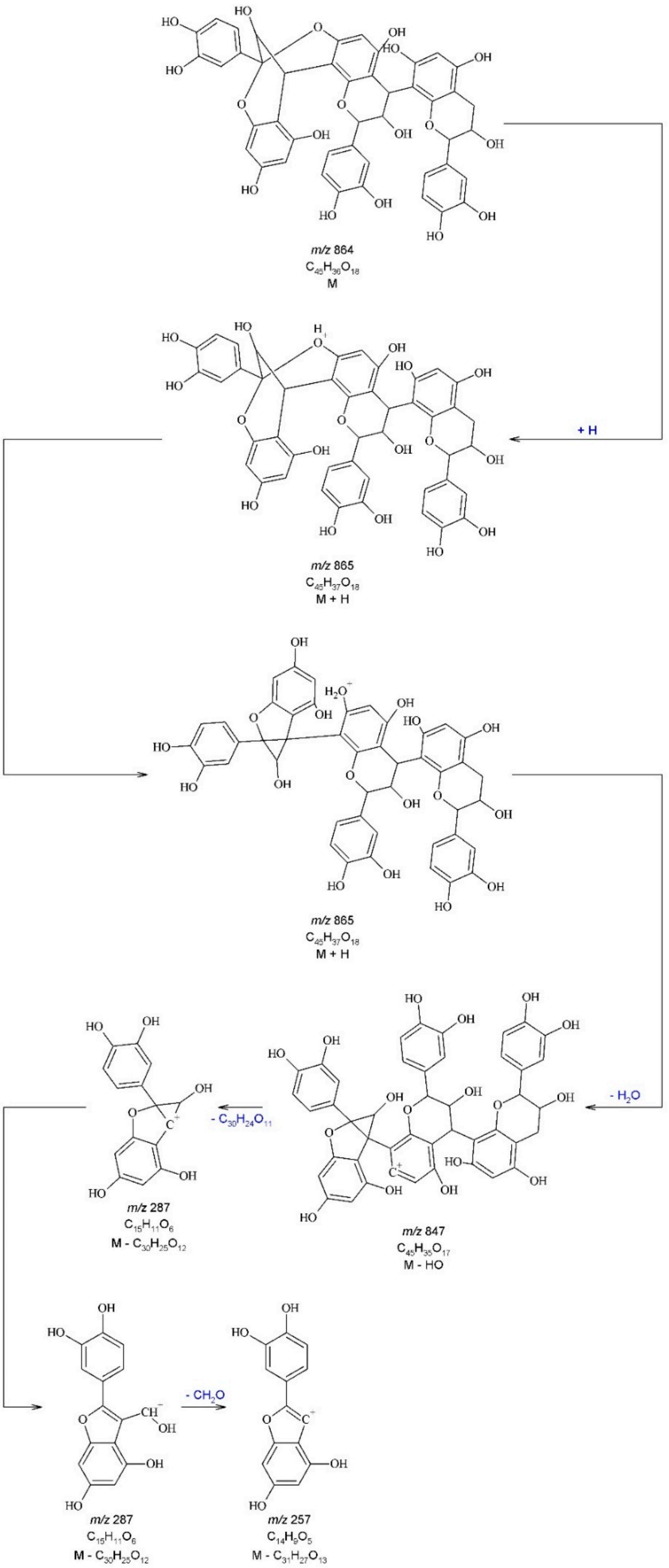

4. Fragmentation Pathway of Lindetannin leading to product ions at m/z 287

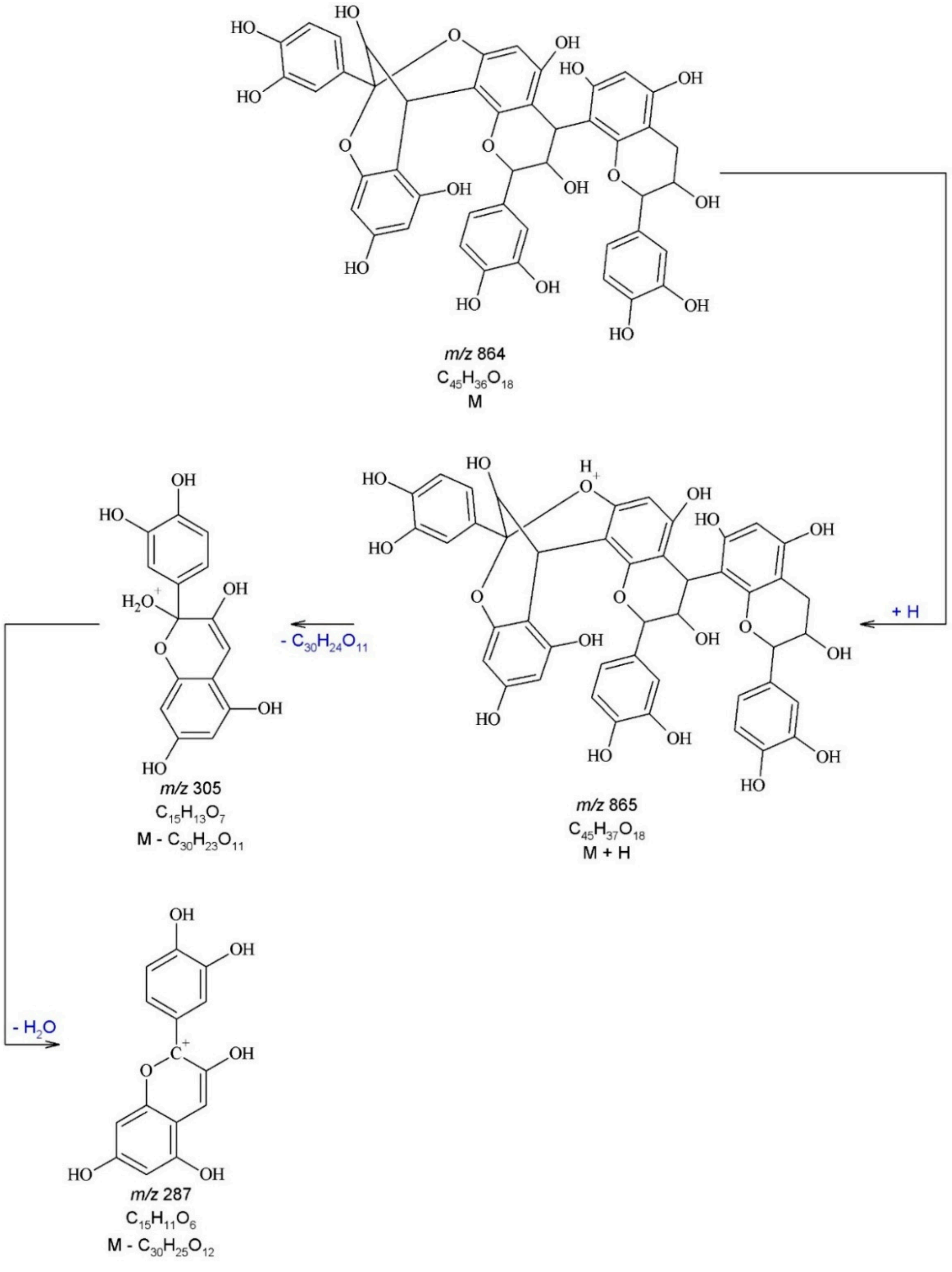

Supplement: Supplementary file 1 [file molecules-31-03233-s001.zip › molecules-4501184-supplementary.pdf]
